# Assessments of patients' Experiences of breathlessness during a spontaneous breathing trial

**DOI:** 10.1186/2197-425X-3-S1-A101

**Published:** 2015-10-01

**Authors:** HS Haugdahl, SL Storli, B Meland, K Dybwik, U Romild, P Klepstad

**Affiliations:** Levanger Hospital, Levanger, Norway; UiT The Artic University of Norway, Tromsø, Tromsø Norway; Nord Trøndelag University College, Levanger, Norway; St.Olav University Hospital, Trondheim, Norway; University of Nordland, Bodø, Norway; Nordland Hospital, Bodø, Norway; Public Health Agency of Sweden, Østersund, Sweden; Norwegian University of Science and Technology, Trondheim, Norway

## Introduction

Breathlessness is a prevalent and distressing symptom in intensive care unit patients. Patients' perception of breathing during a spontaneous breathing trial (SBT) might be correlated to extubation success. There is little evidence of the ability of health care workers to assess the patients' experiences of breathing.

## Objectives

To assess mechanically ventilated patients' experiences of breathlessness during SBT.

## Methods

A prospective observational multicenter study of 100 mechanically ventilated patients. We assessed the agreement between nurses, physicians and patients' scores of breathlessness, perception of feeling secure and improvement of respiratory function at the end of a SBT. We also determined the association between breathlessness and demographic factors or respiratory observations. Self-reported breathlessness, feeling secure and improvement of respiratory function was reported at the end of a SBT by 11-point Numerical Rating Scales.

## Results

Sixty-two patients (62%) reported moderate or severe breathlessness. The median intensity of breathlessness reported by the patients was 5 compared to 2 by nurses and physicians (p < 0.001). Patients felt less secure and reported less improvement of respiratory function compared to nurses and physicians ratings. About half of the nurses and physicians underestimated breathlessness (difference score ≤ -2) compared to the patients´ self-reports. Underestimation of breathlessness was not associated with professional competencies, whether the nurse or physician was involved in previous patient care or number of years working in an intensive care unit. Breathlessness was not related to objective assessments of respiratory function.

## Conclusions

Patients reported higher breathlessness after SBT compared to nurses and physicians. The data suggests that patients' self-report of breathlessness should if possible be included in the evaluation of a SBT.

## Grant

The study was supported by a grant from the Norwegian Nurses Organisation. The funder had no role in the design and conduct of the study; collection, management, analysis and interpretation of the data.
